# RnBeads 2.0: comprehensive analysis of DNA methylation data

**DOI:** 10.1186/s13059-019-1664-9

**Published:** 2019-03-14

**Authors:** Fabian Müller, Michael Scherer, Yassen Assenov, Pavlo Lutsik, Jörn Walter, Thomas Lengauer, Christoph Bock

**Affiliations:** 1 Max Planck Institute for Informatics, Saarland Informatics Campus, 66123 Saarbrücken, Germany; 2 Graduate School of Computer Science, Saarland University, Saarland Informatics Campus, 66123 Saarbrücken, Germany; 3 Division of Epigenomics and Cancer Risk Factors, German Cancer Research Center (DKFZ), 69120 Heidelberg, Germany; 4 Department of Genetics/Epigenetics, Saarland University, 66123 Saarbrücken, Germany; 5 CeMM Research Center for Molecular Medicine of the Austrian Academy of Sciences, 1090 Vienna, Austria; 6 Department of Laboratory Medicine, Medical University of Vienna, 1090 Vienna, Austria; 7 Present Address: Department of Genetics, Stanford University School of Medicine, Stanford, CA 94305 USA

**Keywords:** DNA methylation, Computational epigenetics, Epigenome-wide association studies, Bisulfite sequencing, Epigenotyping microarrays, Integrative data analysis, Bioinformatics software tool, R/Bioconductor package

## Abstract

DNA methylation is a widely investigated epigenetic mark with important roles in development and disease. High-throughput assays enable genome-scale DNA methylation analysis in large numbers of samples. Here, we describe a new version of our RnBeads software - an R/Bioconductor package that implements start-to-finish analysis workflows for Infinium microarrays and various types of bisulfite sequencing. RnBeads 2.0 (https://rnbeads.org/) provides additional data types and analysis methods, new functionality for interpreting DNA methylation differences, improved usability with a novel graphical user interface, and better use of computational resources. We demonstrate RnBeads 2.0 in four re-runnable use cases focusing on cell differentiation and cancer.

**Electronic supplementary material:**

The online version of this article (10.1186/s13059-019-1664-9) contains supplementary material, which is available to authorized users.

## Background

DNA methylation at CpG dinucleotides is a widely studied epigenetic mark that is involved in the regulation of cell state and relevant for a broad range of diseases. Changes in DNA methylation at promoters and enhancers have been associated with cell differentiation, developmental processes, cancer development, and regulation of the immune system. The vast majority of current assays for DNA methylation profiling use bisulfite treatment to selectively convert unmethylated cytosines (including 5-formyl-cytosine and 5-carboxy-cytosine) into uracil (which is subsequently replaced by thymine), while methylated cytosines (including 5-hydroxy-cytosine) remain unconverted. Bisulfite conversion thus transforms DNA methylation information into DNA sequence information that can be read by next-generation sequencing or DNA microarrays [1, 2].

Whole-genome bisulfite sequencing (WGBS) constitutes the current gold standard for DNA methylation profiling, given its genome-wide coverage and single-basepair resolution [3]. However, WGBS requires deep sequencing of the entire genome (which is a significant cost factor), while shallow sequencing leads to poor sensitivity for detecting small differences in DNA methylation. Reduced representation bisulfite sequencing (RRBS) offers a cost-effective alternative for profiling large sets of patient samples, by focusing the sequencing on a subset of the genome enriched using restriction enzymes [4]. RRBS is particularly useful for studying DNA methylation heterogeneity, which profits from deep sequencing coverage and from analyzing many samples using a sequencing-based assay [5–8]. Target-capture bisulfite sequencing enables the analysis of a defined set of genomic regions in large numbers of samples at low cost per sample, but with high setup cost [9, 10]. Finally, the microarray-based Infinium DNA methylation assays—including the MethylationEPIC BeadChip (EPIC) and its predecessor, the HumanMethylation450 BeadChip (450k)—facilitate standardized, high-throughput DNA methylation profiling of a pre-defined subset of CpGs in large sample cohorts [11, 12].

These assays have enabled DNA methylation mapping for a large number of cell types [13, 14] and, following the concept of epigenome-wide association studies (EWAS), for various diseases with a suspected role of epigenetic regulation [15, 16]. The resulting datasets typically comprise DNA methylation profiles as well as sample annotations such as tissue or cell type, phenotypic data (donor age, sex, etc.), and sample grouping (case vs. control, treated vs. untreated, etc.). The primary goal for the bioinformatic analysis of such datasets is to identify characteristic and reliable DNA methylation patterns, and to associate them with relevant annotation data. While various software packages exist that support individual steps of the DNA methylation analysis (reviewed in [17–20] and summarized as a feature table in Additional file 1: Table S1), many users would benefit from an integrative analysis tool that provides extensive, easy-to-understand analysis reports and that requires minimal configuration and no detailed bioinformatic knowledge of the various analysis steps.

We have previously developed the RnBeads software package [21] as a start-to-finish pipeline for DNA methylation analysis in accordance with established standards and practices [16–18]. Initially released in 2012 and published in 2014 [21], RnBeads has become a popular and widely used tool for DNA methylation analysis (200–300 downloads per month from Bioconductor). Here, we present a new, substantially extended version of RnBeads, providing an up-to-date, user-friendly, feature-rich, and readily scalable workflow for the bioinformatic analysis of DNA methylation datasets. The new RnBeads 2.0 software package addresses feedback and feature requests from the tool’s active user community, implements new analysis methods, introduces a graphical user interface, and improves computational efficiency. With these advances, RnBeads provides state-of-the-art support for DNA methylation data analysis in an easy-to-use way, with high flexibility and performance.

## Results and discussion

### RnBeads overview and new features

**Fig. 1:**
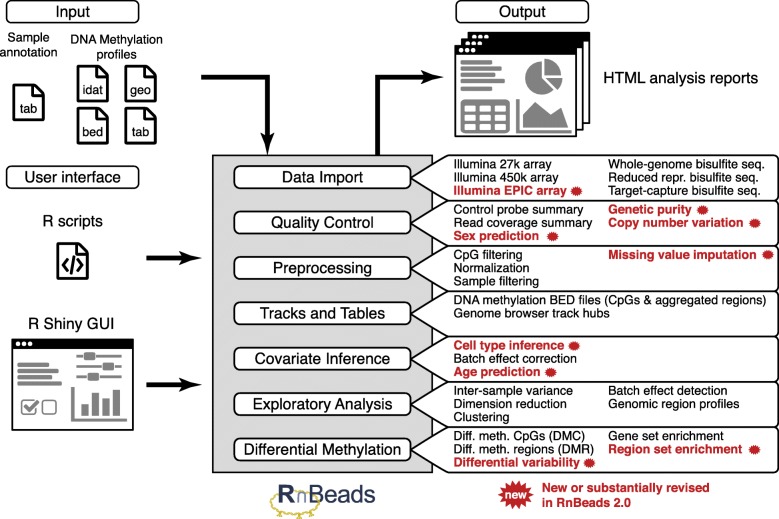
Overview of the RnBeads analysis workflow and new features added in RnBeads 2.0. Conceptual drawing of the RnBeads workflow for DNA methylation analysis, listing key features (right) for each of the RnBeads analysis modules (center), with newly added features indicated in bold red text. tab, tabular (e.g., comma-separated) files; idat, Infinium signal intensity files; geo, download from the GEO data repository

RnBeads includes modules for data import, quality control, filtering and normalization (“preprocessing”), export of processed data (“tracks and tables”), covariate inference (e.g., predicting epigenetic age and cell type heterogeneity from DNA methylation data), exploratory analysis (e.g., dimension reduction, global distribution of DNA methylation levels, hierarchical clustering), and differential DNA methylation analysis (Fig. 1). Each analysis module generates an HTML report that combines method descriptions, results tables, and publication-grade plots. These reports provide the user with a comprehensive and readily sharable summary of the dataset.

Of the various features that we introduced into RnBeads since the original publication in 2014, we specifically highlight the following four areas:

1. *New data types and cross-platform analysis:* RnBeads now supports EPIC microarrays and enables seamless data integration across different DNA methylation assays (e.g., EPIC, 450k, and 27k microarrays as well as WGBS and RRBS), which facilitates DNA methylation meta-analyses that combine several data sources into a single set of results.
2. *Extended analysis and inference methods:* We added new functionality for handling incomplete data and missing values, for detecting genetic evidence of sample contamination or low data quality, for quantifying DNA methylation heterogeneity, and for DNA methylation-based inference of phenotypic information. We incorporated the LUMP algorithm [22], which estimates immune cell content of tumors and other heterogeneous tissue samples, and epigenetic age prediction [23] for both Infinium microarray and bisulfite sequencing data. These predictions are useful not only for inferring missing donor annotations, but also for detecting deviations indicative of accelerated aging [24] or evidence of sample mix-ups. Additional new features include the identification of genomic regions characterized by differential DNA methylation variability [25, 26] and genomic region enrichment analysis using the LOLA tool [27].
3. *New user-friendly interface:* We provide a graphical user interface for RnBeads that facilitates the configuration and execution of DNA methylation analyses. Together with RnBeads’ interactive and self-explanatory HTML reports, this new interface makes RnBeads analyses more readily accessible for users with limited R/Bioconductor knowledge.
4. *Improved computational efficiency:* Using parallelization and automatic distribution of RnBeads analyses across a high-performance computing (HPC) cluster, we were able to process datasets comprising hundreds of RRBS/WGBS profiles and thousands of microarray-based profiles in a single analysis run.

To illustrate the practical utility of these new RnBeads features, we present four use cases: (i) DNA methylation in human peripheral blood samples, (ii) cell type-specific DNA methylation in human hematopoiesis, (iii) DNA methylation heterogeneity in cancer samples, and (iv) cross-platform DNA methylation analysis. Detailed re-runnable versions of these analyses, including configurations and results, are available for visualization and download from the RnBeads website (https://rnbeads.org/methylomes.html). These pre-configured analyses and pre-calculated reports also provide a good starting point for learning about the use of RnBeads, thereby complementing the tutorials provided on the RnBeads website (https://rnbeads.org/tutorial.html), and for configuring custom analyses that integrate newly generated datasets with publicly available reference data.

### Use case 1: Analyzing DNA methylation in a large cohort of peripheral blood samples

**Fig. 2:**
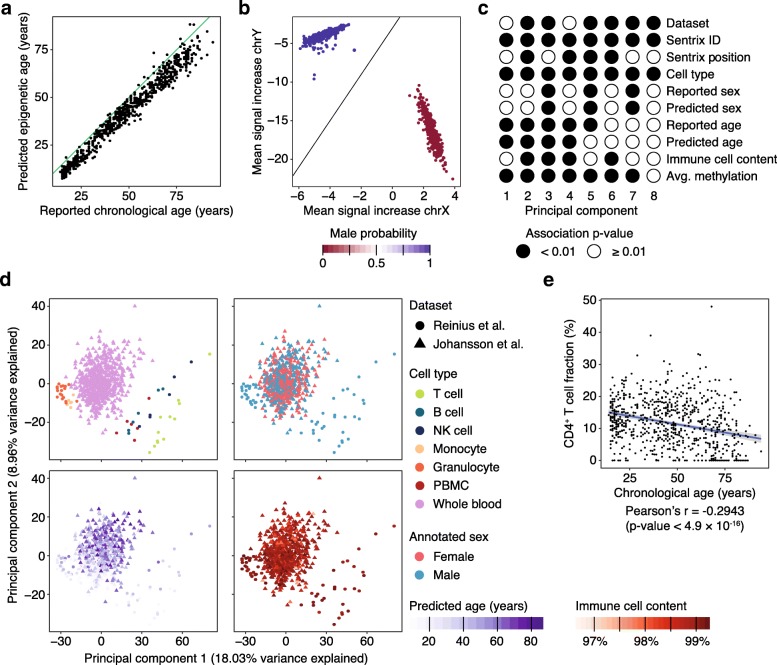
Analysis of a large DNA methylation dataset of blood samples profiled using Infinium 450k. **a** Scatterplot showing the correlation between epigenetic age predicted from DNA methylation and reported chronological age for 729 healthy donors (three individuals were excluded because no chronological age was reported). **b** Positioning of the samples in two-dimensional space for sex prediction. **c** Statistical association between principal components (columns) and sample annotations (rows). Significant associations with *p* values below 0.01 are marked by filled circles, while non-significant values are represented as empty circles. **d** Principal component analysis for 792 blood-based DNA methylation profiles, comprising 732 peripheral blood samples and 60 sorted blood cell populations, using the same principal components as in panel **c**. Immune cell content was estimated using the LUMP algorithm. **e** Scatterplot showing the negative correlation between chronological age and the estimated fraction of CD4^+^ T cells

To illustrate the use of RnBeads for analyzing DNA methylation microarray data in a large cohort, we obtained Infinium 450k profiles for peripheral blood samples of 732 healthy individuals [28]. We also included reference profiles for sorted blood cell types [29], in order to account for inter-individual differences in the frequency of different cell types [30]. First, we used RnBeads to infer donor age and sex for each sample, thereby filling in a handful of missing annotations with imputed values, while also checking for potential sample mix-ups among those samples that have donor age and sex documented as part of their annotation (Fig. 2a, b). Second, we performed reference-based estimation of immune cell composition [30] as implemented in RnBeads, and we found that the inferred immune cell content [22] (as well as other annotations) are indeed associated with important principal components of the DNA methylation dataset (Fig. 2c, d). Our results emphasize the need to correct for these covariates when identifying CpGs and genomic regions that are associated with the annotation(s) of primary interest. Third, we compared chronological age with the fraction of CD4^+^ T cells inferred from the DNA methylation data using sorted blood cell types as reference [30] (Fig. 2e) and observed a negative correlation, consistent with the known age-related shift toward myeloid (instead of lymphoid) hematopoiesis [31]. In summary, this use case illustrates the prediction of age, sex, and cell composition based on DNA methylation data, and it provides a framework for microarray-based epigenome-wide association studies.

### Use case 2: Dissecting the DNA methylation landscape of human hematopoiesis

**Fig. 3:**
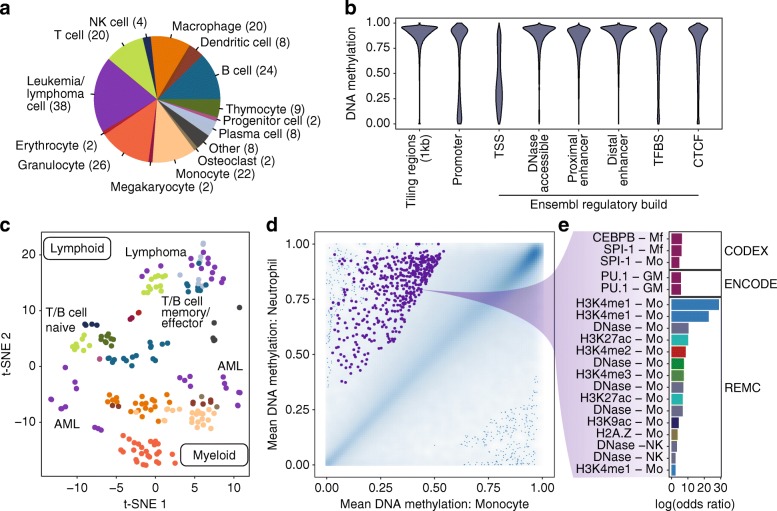
Genome-wide analysis of DNA methylation in hematopoietic cells profiled using WGBS. **a** Overview of cell types and sample numbers in the BLUEPRINT WGBS dataset (August 2016 release), which was analyzed with RnBeads. **b** Distribution of DNA methylation levels for different types of genomic region sets. **c** t-SNE dimension reduction based on Euclidean distances of mean DNA methylation in putative regulatory regions. Cell types are color-coded as in panel **a**. **d** Density scatterplots showing differential DNA methylation levels between monocytes (*N* = 20) and neutrophils (*N* = 10). Point density is indicated by blue shading. The 0.1% of regions in the most sparsely populated areas of the plot are shown as individual points. The 500 highest-ranking hypomethylated regions in monocytes compared to neutrophils are indicated in purple. **e** Log-odds ratios of the LOLA enrichment analysis for the 500 regions highlighted in panel **d**. The top 20 most enriched categories of the LOLA Core and Extended databases are shown. Differently colored bars represent different types of genomic region data (e.g., peaks for histone marks or transcription factor binding sites). Mf, macrophage; GM, lymphoblastoid cell; Mo, monocyte; REMC, Roadmap Epigenomics Mapping Consortium

The efforts of the International Human Epigenome Consortium [14] and its contributing projects have resulted in large sets of publicly available WGBS data for various cell types. To demonstrate RnBeads’ ability to process such large reference collections, we analyzed a DNA methylome dataset comprising 195 WGBS profiles and 26,238,599 unique CpG sites (after the preprocessing step) for various hematopoietic cell types (Fig. 3a), which was originally established by the BLUEPRINT project [32]. Focusing on pre-defined genomic region sets including the Ensembl Regulatory Build [33], we observed the expected distribution of DNA methylation, with high levels of DNA methylation in genome-wide tiling regions, slightly lower levels at enhancers and transcription factor binding sites, and much lower levels (and a bimodal distribution) of DNA methylation at gene promoters and transcription start sites (Fig. 3b). The DNA methylation profiles clustered according to cellular lineage (lymphoid vs. myeloid cells), cell maturation stage (naïve vs. effector/memory cells), and cell type (Fig. 3c). Comparing two myeloid cell types (monocytes and neutrophils), RnBeads identified decreased DNA methylation levels in monocytes at a subset of putative regulatory regions (Fig. 3d). LOLA analysis for enrichment of genomic region sets [27] (a new feature we introduced in RnBeads 2.0 to facilitate biological interpretation) identified characteristic enrichment for cell type-specific regulatory regions (including monocyte-specific open chromatin and its associated histone modifications) and for the binding sites of important hematopoietic transcription factors such as CEBPB and SPI-1/PU.1. In summary, this use case demonstrates the scalability of RnBeads to large DNA methylation datasets (which involves the distribution of analysis jobs across an HPC cluster for efficient parallelized calculation), region-based analysis of DNA methylation, and biological interpretation by region set enrichment analysis.

### Use case 3: Quantifying DNA methylation heterogeneity in a childhood cancer cohort

**Fig. 4:**
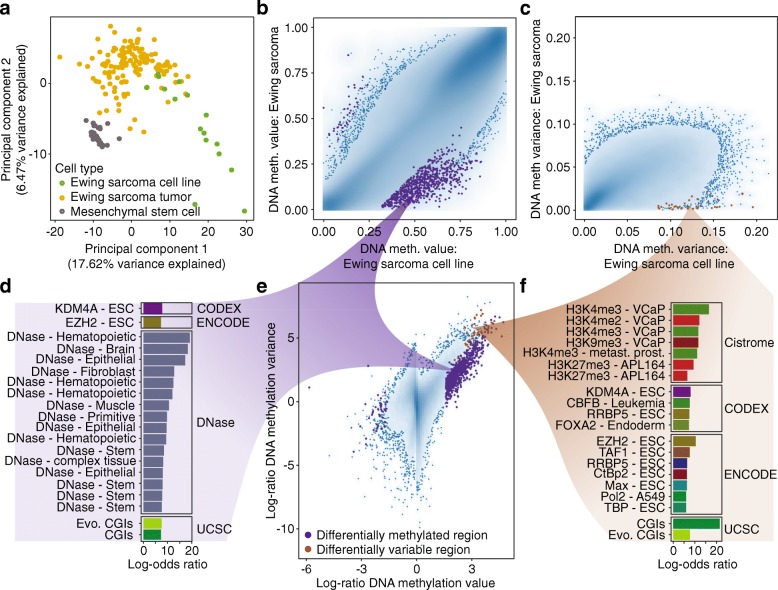
Dissection of DNA methylation heterogeneity in Ewing sarcoma samples profiled using RRBS. **a** Principal component analysis of an RRBS dataset for Ewing sarcoma tumors, cell lines, and mesenchymal stem cells, based on DNA methylation values aggregated across Ensembl Regulatory Build regions. **b** Density scatterplot comparing the aggregated DNA methylation levels between Ewing sarcoma tumors (*N* = 140) and Ewing sarcoma cell lines (*N* = 16). Marked in purple are the most highly ranking differentially methylated regions up to an automatically selected rank cutoff. **c** Density scatterplot comparing DNA methylation variance between Ewing sarcoma tumors and cell lines. Significant differentially variable regions are marked in brown. **d** Enrichment (log-odds ratios) based on LOLA analysis for the differentially methylated regions shown in panel **b** and in panel **e**. Differently colored bars represent different types of genomic region data. **e** Density scatterplot comparing the log-ratios between DNA methylation levels and variance in Ewing sarcoma tumors and cell lines. **f** Enrichment (log-odds ratios) based on LOLA analysis for differentially variable regions shown in panel **c** and in panel **e**. ESC, embryonic stem cell; CGIs, CpG islands

Epigenetic heterogeneity has recently emerged as a key property of tumor samples [34]. To demonstrate the utility of RnBeads for cancer research, we re-analyzed 188 recently published RRBS profiles of Ewing sarcoma tumors, cell lines, and mesenchymal stem cells [7]. Ewing sarcoma is a pediatric bone cancer characterized by low genetic heterogeneity but striking changes in the epigenome [7, 35]. Data processing and quality control resulted in 2,217,186 unique CpG sites that were covered by at least five sequencing reads in more than 50% of the samples. Based on these CpGs, we aggregated DNA methylation values in each sample across annotated genomic regions, including putative regulatory elements defined in the Ensembl Regulatory Build [33]. Principal component analysis showed the expected separation between tumors, cell lines, and mesenchymal stem cells, with higher sample-to-sample heterogeneity among the tumors and cell lines compared to mesenchymal stem cells (Fig. 4a). We compared the primary tumors with the cell lines using the differential DNA methylation module of RnBeads, and we found that most of the differentially methylated regions were hypermethylated in the cell lines (Fig. 4b). We also observed increased variance in the cell lines (Fig. 4c). LOLA analysis detected markedly different enrichments among differentially methylated regions (DMRs) and differentially variable regions (DVRs), indicating that the two measures provide complementary information about the DNA methylation landscape (Fig. 4d–f). Regions hypermethylated in Ewing sarcoma cell lines were enriched for DNase hypersensitive sites in various healthy tissue samples (Fig. 4d), consistent with the widespread hypermethylation and silencing of non-essential regulatory regions in cell lines. In contrast, hypervariable regions were enriched for transcription factor binding and histone modifications in cancer cell lines and embryonic stem cells (Fig. 4f), indicative of increased regulatory plasticity of the Ewing sarcoma cell lines compared to the primary tumors. In summary, this use case describes the analysis of an RRBS-based dataset (which benefits from region-based analysis due to fluctuations in single-CpG coverage), and it demonstrates the utility of RRBS and RnBeads for investigating DNA methylation heterogeneity in tumor samples.

### Use case 4: Analyzing DNA methylation data across different assay platforms

**Fig. 5:**
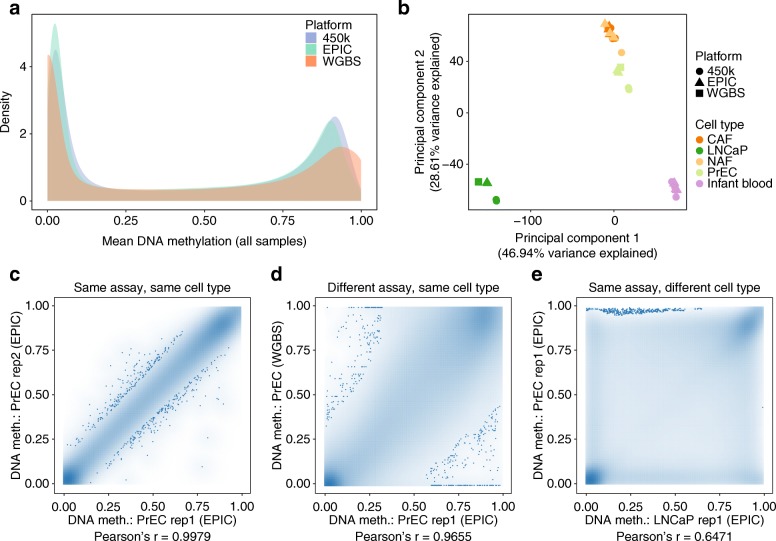
Cross-platform data integration using RnBeads. **a** Distribution of DNA methylation levels for the same samples profiled across different assay platforms. **b** Principal component analysis of the assay comparison dataset. Point shapes and colors depict assay platforms and cell types, respectively. **c–e** Density scatterplots comparing replicates of prostate epithelial cells profiled using the EPIC assay (panel **c**); prostate epithelial cells profiled using the EPIC assay and WGBS (panel **d**); and prostate epithelial cells as well as a prostate cancer cell line profiled with the EPIC assay (panel **e**). Point density is indicated by blue shading. The 0.1% of CpGs in the most sparsely populated areas of the plot are shown as individual points. All plots are based on CpGs that were covered by all three assay platforms. Pearson correlation coefficients are shown below each diagram. NAF, non-malignant tissue-associated fibroblasts; CAF, cancer-associated fibroblasts; PrEC, prostate epithelial cells; LNCaP, prostate cancer cell line

Several generations of Infinium DNA methylation microarrays have been used over the years, and it can be necessary to combine multiple datasets in an integrative analysis. RnBeads now provides dedicated methods for cross-platform analysis, making it possible to combine RnBeads data objects across the different versions of the Infinium microarray (27k, 450k, EPIC) and with bisulfite sequencing data (RRBS, WGBS). To demonstrate this feature, we analyzed a benchmarking dataset comprising three different assay platforms: Infinium 450k microarrays, Infinium EPIC microarrays, and WGBS [36]. All three datasets were loaded and preprocessed separately using RnBeads, which resulted in data objects with 443,053 (450k), 801,716 (EPIC), and 25,918,426 (WGBS) unique CpG sites, respectively. Applying the RnBeads method for combining datasets with the option of including only CpGs covered by all three platforms, these objects were merged into a combined dataset comprising 408,621 shared CpGs. This combined dataset was processed using the RnBeads analysis modules. We observed differences in the global distribution of DNA methylation levels between assays (Fig. 5a). Nevertheless, the principal component analysis showed that the biological differences between samples dominated over the technical differences between platforms (Fig. 5b). Focusing specifically on the comparison between a prostate cancer cell line (LNCaP) and prostate epithelial cells (PrECs), we observed the highest correlation between replicates for the same assay in the same cell type (Pearson’s *r* = 0.9979, Fig. 5c). Nevertheless, the correlation between different assays in the same cell type (Pearson’s *r* = 0.9655, Fig. 5d) was still high and much stronger than the correlation between different cell types for the same assay (Pearson’s *r* = 0.6471, Fig. 5e). In summary, this use case highlights the feasibility and practical utility of cross-platform analysis of DNA methylation using RnBeads.

### Comparison to other software tools for DNA methylation analysis

To assess the computational efficiency of RnBeads, we compared its performance to that of other software packages for DNA methylation analysis [37–40], separately for DNA methylation microarray data, RRBS data, and WGBS data (see the “Methods” section for details and Additional file 2: Table S2 for tool configurations). Given that the different tools provide vastly different feature sets, we considered three scenarios: (i) data import only, (ii) core modules, and (iii) comprehensive analysis with most features activated (Additional file 3: Figure S1). RnBeads was the only tool that supported both microarray-based and bisulfite sequencing-based analysis. For microarray-based analysis, the low-level data processing packages minfi, methylumi, and wateRmelon were faster than ChAMP and RnBeads (which need to prepare the dataset for their more extensive downstream analyses). Compared to ChAMP, RnBeads was more memory-efficient and faster in the comprehensive setting. For bisulfite sequencing-based analysis, RnBeads showed better performance than methylKit on the WGBS dataset in the core module setting, but somewhat longer runtime and higher memory usage on the RRBS dataset. These differences can be attributed to the reformatting into memory-efficient data structures that RnBeads performs during data import. In summary, the runtime performance of RnBeads was similar to that of other tools with more limited functionality, suggesting that the choice of the most suitable tool for DNA methylation analysis depends mainly on the desired features and analysis modes. To assist with an informed selection, we thus surveyed a broad range of tools for DNA methylation analysis and assembled a detailed feature table based on the tool documentations (Additional file 1: Table S1). RnBeads emerged from this comparison as the software that implements the most comprehensive workflow for analyzing DNA methylation data, while also providing a user-friendly interface and extensive options for reporting and reproducibility.

## Conclusions

RnBeads is an integrated software package for the analysis and interpretation of DNA methylation data. Due to its modular design and optional graphical user interface, the software is well suited for both beginners and experts in the field of DNA methylation analysis. Interactive reports provide a comprehensive overview of DNA methylation datasets while also fostering reproducibility (by documenting parameter settings) and robustness (by making it easy to evaluate different parameter settings). RnBeads 2.0 implements various new features that arose from technological advances and valuable user feedback. For example, support for the Infinium EPIC assay and for cross-platform data integration broaden the scope of RnBeads analyses; new and improved methods for the DNA methylation-based prediction of age and sex are useful for large cohort studies; a graphical user interface makes many RnBeads features more easily accessible; the analysis of DNA methylation variability adds a new dimension to epigenome-wide association studies; and LOLA-based region set enrichment analysis facilitates the biological interpretation of DNA methylation differences. RnBeads also establishes a convenient way of interfacing with reference epigenome datasets and integrating them into custom analyses, as demonstrated by the integration of sorted blood cell profiles in our first use case. We processed several such datasets and provide re-runnable analyses on the website (https://rnbeads.org/methylomes.html). The presented use cases provide concrete examples for RnBeads analysis of DNA methylation and illustrate some of the tool’s key features. Typical applications of RnBeads include epigenome-wide association studies, reference epigenome analysis, investigation of cancer heterogeneity, and epigenetic biomarker development.

## Methods

### Installing RnBeads

RnBeads is implemented in R/Bioconductor and can be installed in the usual way for Bioconductor packages [41]. Furthermore, the “Installation” section on the RnBeads website (https://rnbeads.org/installation.html) provides an RnBeads installation script, which validates that all package dependencies are installed and up to date. The FAQ page on the RnBeads website (https://rnbeads.org/faq.html) addresses typical problems and answers common questions. Finally, users can contact the RnBeads developers (team@rnbeads.org) for further information and assistance.

### Running RnBeads

RnBeads supports any genome-wide or genome-scale assay that provides quantitative DNA methylation data at single-CpG resolution. This includes Infinium DNA methylation microarrays (27k, 450k, EPIC) and bisulfite sequencing protocols (WGBS, RRBS, etc.). Data obtained using enrichment-based assays (e.g., MeDIP-seq, MBD-seq, MRE-seq) can also be processed after their read-count output has been converted to single-CpG methylation levels using bioinformatic inference tools [42–45]. We currently provide RnBeads annotation packages for the human, mouse, and rat genomes. In addition, users can prepare customized annotation packages for their species of interest, using the RnBeadsAnnotationCreator package (https://rnbeads.org/tutorial.html). To start an RnBeads analysis, the user provides a sample annotation table as well as the DNA methylation data. For microarray-based analyses, RnBeads accepts raw signal intensity files (IDAT) as well as preprocessed DNA methylation data in tabular form. Bisulfite sequencing data are imported directly from the output of DNA methylation calling software such as Bis-SNP or Bismark [46, 47], or as tabular text files providing genomic coordinates of individual CpGs together with their DNA methylation levels and read coverage. Finally, RnBeads can import DNA methylation data directly from the Gene Expression Omnibus (GEO) data portal.

### Graphical user interface

A key feature of RnBeads is its self-configuring workflow, which supports launching of a full DNA methylation analysis with a single command (rnb.run.analysis(…)) on the R command line [21]. However, some types of analysis may require more extensive configuration, for which RnBeads includes an extensive set of customizable parameters (which can be specified in R or imported from XML configuration files). Moreover, RnBeads provides various R functions for operating directly on the RnBSet R data objects used to store and process DNA methylation data and associated sample annotations in RnBeads. To make customized RnBeads analysis easier to configure, we have developed RnBeadsDJ, a graphical user interface for RnBeads that is based on the R Shiny toolkit (http://shiny.rstudio.com/). This interface allows the user to launch RnBeads through a web browser and interactively specify the input data and analysis options. The user can either launch the complete analysis pipeline or execute modules individually. A detailed tutorial for RnBeadsDJ is available on the RnBeads website (https://rnbeads.org/tutorial.html).

### Age prediction

DNA methylation patterns have been linked to human aging and can be used to infer the chronological age of healthy individuals [23, 48]. Furthermore, the difference between (predicted) epigenetic age and (known) chronological age appears to reflect the speed of biological aging in a way that is predictive of various health issues [49]. We have incorporated the DNA methylated-based prediction of epigenetic age into RnBeads, using elastic net regression at the single-CpG level (https://rnbeads.org/ageprediction.html). RnBeads includes pre-trained models for age prediction as well as the option for users to provide their own training data. It supports age inference based on both DNA methylation microarrays and bisulfite sequencing datasets.

### Sex prediction

RnBeads uses differences in copy number of genomic regions located on the sex chromosomes to predict the sex of sample donors, in order to infer missing annotation information or to detect sample mix-ups. For microarray data, sex prediction is based on the comparison of the average signal intensities for the sex chromosomes with those on the autosomes, calculating a predicted sex probability by logistic regression. For bisulfite sequencing data, RnBeads compares the sequencing coverage for the sex chromosomes with those for the autosomes, followed by logistic regression trained on datasets with known sex information.

### Region-based analysis

In addition to analysis based on individual CpGs, RnBeads aggregates and compares DNA methylation levels across genomic regions of interest, which can enhance statistical power and interpretability [17]. Extending the default region sets available in RnBeads (genes and gene promoters, CpG islands, and genomic tiling regions), the RnBeads website now provides a collection of additional region sets for automatic import (https://rnbeads.org/regions.html). This collection includes region sets defined based on consensus epigenome profiles such as putative regulatory regions in the Ensembl Regulatory Build [33] and regions associated with DNA methylation variability [50].

### Missing values

The presence of missing values in DNA methylation datasets constitutes an important analytical challenge, for which RnBeads implements several alternative solutions, namely: Sample-wise means and medians, CpG-wise means and medians, random replacement from other samples in the dataset, and k-nearest neighbor (KNN) imputation. KNN imputation tends to provide adequate estimates of missing values when enough nearby data points are available. It has been used extensively for gene expression microarray data [51], and it has also been applied to DNA methylation data [8]. For those cases in which the model assumptions of KNN imputation are not met due to disproportionally high numbers of missing values (which is not uncommon for bisulfite sequencing datasets), we implemented the mean and median imputation approaches.

### Genetic purity

For the Infinium microarrays, RnBeads implements a new metric of sample quality that we call “genetic noise”. This metric quantifies the deviation of the signals of autosomal single nucleotide polymorphism probes on the microarray from the expected values of 0 and 1 (homozygosity) as well as 0.5 (heterozygosity) of a diploid cell. Such deviations can indicate technical problems of the microarray-based analysis, contamination with DNA samples from other individuals, or deviations from the diploid case (e.g., aneuploid cancer samples).

### Cell type heterogeneity

RnBeads implements reference-based and reference-free methods for estimating intra-sample heterogeneity [21, 30, 52, 53]. This includes reference-based estimation of immune cell content [30] based on the DNA methylation profiles of purified blood cell populations [29] as well as the LUMP algorithm [22] for estimating immune cell invasion in bulk tumor samples based on a preselected set of CpGs that are exclusively unmethylated in blood cells. While this algorithm was developed specifically for the Infinium 450k assay, its implementation in RnBeads supports both microarray-based and bisulfite sequencing-based assays.

### Differential variability

CpGs and genomic regions can differ between cases and controls not only in terms of their average DNA methylation levels, but also in terms of the variability of DNA methylation levels; for example, epigenetic variability may be higher or lower in tumors than in healthy tissue. In RnBeads, users can choose between two algorithms to quantify differential variability: diffVar [25] and iEVORA [26]. DiffVar uses an empirical Bayes framework, while iEVORA is based on the Bartlett test, which tests for differences in variance (heteroscedasticity) across samples. Striking the right balance between reporting too few and too many differentially variable cytosines (DVCs) and differentially variable regions (DVRs) represents an unsolved statistical challenge, especially when the data do not follow a normal distribution. Therefore, we implemented a strategy analogous to the identification of differentially methylated cytosines (DMCs) and differentially methylated regions (DMRs) between sample groups in RnBeads: DVCs and DVRs are ranked by the worst (highest) rank of the following criteria: (i) the adjusted *p* value of the statistical test (either diffVar or iEVORA), (ii) the difference in variance between the groups, and (iii) the log-ratio of the two group-wise variances. RnBeads produces summary plots comparing group-wise variances, *p* values, and ranks, while also exporting detailed tables of DVCs and DVRs.

### Enrichment analysis

To investigate the biological processes relevant to observed DNA methylation differences, RnBeads implements region set enrichment analysis using LOLA [27], in addition to gene set analysis based on Gene Ontology terms. The LOLA tool compares a set of genomic regions of interest (i.e., DMRs and/or DVRs) to a potentially large reference catalogue of region sets using Fisher’s exact test and derives a ranked list of significantly enriched region sets. By default, RnBeads uses the LOLA Core database as a reference, which includes transcription factor binding sites, tissue-specific enhancer elements, and genome annotations such as CpG islands and repetitive elements. Moreover, other LOLA databases such as the LOLA Extended database (http://databio.org/regiondb) or user-created databases can be included via RnBeads option settings. Plots showing enrichment *p* values and log-odds ratios visualize the most enriched region sets in the RnBeads report.

### Computational scalability

We have successfully processed datasets comprising hundreds of RRBS and WGBS samples and thousands of Infinium microarrays with RnBeads. To handle large memory requirements, RnBeads uses disk-based matrices implemented in the ff R package (https://CRAN.R-project.org/package=ff). Tasks are parallelized using the foreach (https://CRAN.R-project.org/package=foreach) and doParallel R packages (https://CRAN.R-project.org/package=doParallel). We have also developed an interface that facilitates the automatic distribution of RnBeads analysis runs across an HPC cluster (e.g., managed through a “grid engine” or “slurm” job scheduler). Finally, to facilitate DNA methylation analysis on small computers including personal laptops, RnBeads provides options that disable the most resource-intensive steps; these configurations are available as pre-defined option profiles for low-, medium-, and high-resource settings.

### Tool comparison

We compared RnBeads in terms of its runtime performance and peak memory consumption with other software packages for DNA methylation microarray analysis highlighted in a recent review paper [54], namely minfi [37], methylumi (http://bioconductor.org/packages/release/bioc/html/methylumi.html), watermelon [38], and ChAMP [39], and with the methyKit [40] package for analyzing bisulfite sequencing data. The microarray-based tools were benchmarked on the first use case (732 blood samples), while the bisulfite sequencing tools were benchmarked on a mouse RRBS dataset (GSE45361, 6 adrenal gland and 11 liver samples) and on a human WGBS dataset (12 hepatocyte samples) from the DEEP project (http://www.deutsches-epigenom-programm.de/), thus covering a broad range of different scenarios for DNA methylation analysis. The benchmarking was performed on a Debian Wheezy machine with 32 cores (1.2 GHz) and 126 GB RAM using R-3.5.0. Three different tool configurations with different depths of analysis were evaluated (Additional file 2: Table S2): (i) data import only, (ii) core modules enabled, and (iii) comprehensive analysis with most features enabled. Furthermore, to complement the performance-oriented benchmarking with a feature-oriented comparison, we conducted a comprehensive survey of popular software tools for DNA methylation analysis in comparison to RnBeads (Additional file 1: Table S1). To that end, we manually reviewed the documentation of all Bioconductor packages for DNA methylation analysis that had a popularity ranking of 900 or better (https://www.bioconductor.org/packages/release/BiocViews.html#___DNAMethylation). We considered only packages that support DNA methylation microarrays and/or bisulfite sequencing data, while discarding data broker packages and packages for single, specialized tasks. We also considered selected DNA methylation analysis tools outside Bioconductor based on the literature review.

## Additional files

Additional file 1: **Table S1.** Feature comparison table of software tools for DNA methylation analysis. (XLSX 30 kb)

Additional file 2: **Table S2.** Parameter settings for the benchmarking of DNA methylation analysis tools. (XLSX 12 kb)

Additional file 3: **Figure S1.** Performance of RnBeads and other DNA methylation analysis tools. (PDF 149 kb)

## Abbreviations

27k: Illumina Infinium HumanMethylation27 BeadChip
450k: Illumina Infinium HumanMethylation450 BeadChip
DMC: Differentially methylated cytosine
DMR: Differentially methylated region
DVC: Differentially variable cytosine
DVR: Differentially variable region
EPIC: Illumina Infinium MethylationEPIC BeadChip
GEO: Gene Expression Omnibus
HPC: High-performance computing
RRBS: Reduced representation bisulfite sequencing
SRA: Sequence Read Archive
WGBS: Whole-genome bisulfite sequencing
