# Supplementary material for: RnBeads 2.0: comprehensive analysis of DNA methylation data
Source: Genome Biol. 2019 Mar 14;20:55. doi: 10.1186/s13059-019-1664-9 (PMC6419383; doi:10.1186/s13059-019-1664-9)
Supplement: Supplementary file 3 — Figure S1. Performance of RnBeads and other DNA methylation analysis tools. (PDF 149 kb) [file 13059_2019_1664_MOESM3_ESM.pdf]

Additional file 3

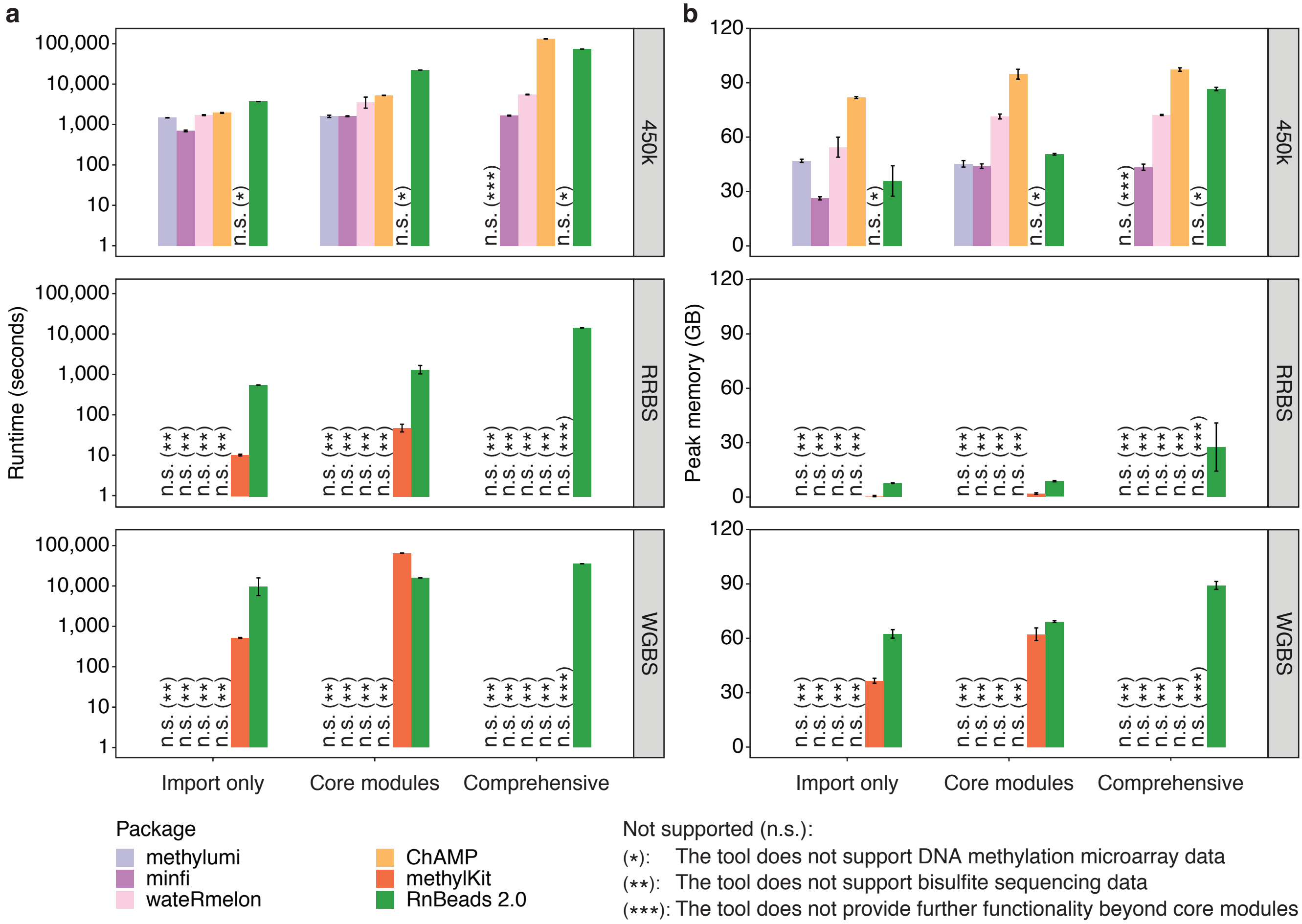

Figure S1. Performance of RnBeads and other DNA methylation analysis packages.

The performance of RnBeads was compared to other R packages for DNA methylation analysis in terms of **(a)** runtime and **(b)** peak memory consumption. The packages were applied to three datasets corresponding to the Infinium 450k, RRBS, and WGBS platforms, and they were run in three different settings (data import only, core modules, comprehensive analysis) as described in the Methods section.
